# Impact of e-service quality on e-loyalty of online banking customers in Pakistan during the Covid-19 pandemic: mediating role of e-satisfaction

**DOI:** 10.1186/s43093-023-00201-8

**Published:** 2023-05-23

**Authors:** Fahad Najeeb Khan, Muhammad Usman Arshad, Muhammad Munir

**Affiliations:** 1 Dongbei University of Finance and Economics, Dalian, China; 2 Department of Commerce, University of Gujrat, Hafiz Hayat Campus, Jalalpur Road, Gujrat, Pakistan; 3 Department of Management and Administrative Sciences, University of Narowal, Narowal, Pakistan

**Keywords:** E-service quality, E-satisfaction, E-loyalty, Online banking, M10, M30, M31

## Abstract

This study explores the mediating role of e-satisfaction during the pandemic on the relationship between e-service quality and e-loyalty of banking customers in Pakistan. The data were collected from 442 customers of online banking services in Pakistan during the Covid-19 pandemic, following a survey-based study. Baron and Kenny (J Personal Soc Psychol, 51(6):1173, 1986) and Preacher and Hayes (Behav Res Methods, 40(3):879-891, 2008) mediation technique which utilizes the bootstrapping method has been used to explore mediation. The findings show that e-service quality has a significant positive effect on the e-loyalty of the customers of online banking services. Relationships between e-service quality and e-loyalty of online banking customers in Pakistan are significantly and fully mediated by their online satisfaction in unusual situations. This study would help the bankers to implement more effective marketing strategies to retain their customers and attract potential customers, particularly during non-normal situations like the Covid-19 pandemic. It will help them identify the areas of e-services that need improvement to enhance the satisfaction and loyalty of the customers. The bootstrap method for mediation along with Baron and Kenny (J Personal Soc Psychol, 51(6):1173, 1986) leads to using a more sophisticated methodological technique to explore the mediation. The Oliver Expectancy-Disconfirmation Paradigm (EDP) in electronic banking setup during non-normal situations like the Covid-19 pandemic also served as a unique contribution to this study. Application of Baron and Kenny (J Personal Soc Psychol, 51(6):1173, 1986) mediation along with Preacher and Hayes (Behav Res Methods, 40(3):879-891, 2008) leads to more robust findings for the study in non-normal situations like the Covid-19 pandemic. The study findings add scientific value as they are applicable to the banking sector in particular in non-normal situations like the Covid-19 pandemic and the overall service sector in general. Further, as two different methods of mediation have been employed and this makes the study more rigorous and scientific.

## Introduction

Over the last 2 decades, the increasing use of the internet has given rise to new trading methods and transactions like e-commerce [1]. Advancement in technology has facilitated both customers and companies in many areas, and one of these is online banking. New Companies, customers and new product types have gained from the expansion of electronic commerce due to the Covid-19 pandemic.^1^ Even there are contact restrictions due to lockdowns for businesses, but customers can shop conveniently and securely from their homes due to this expansion of e-commerce [6].“Online banking uses the internet to carry out banking transactions like funds transfer, bill payments, investments and mobile top-up”, etc., as referred by [18]. These services are offered both via the desktop version and mobile apps. Almost every banking service is provided electronically as the pandemic has forced people to maintain social distancing and avoid personal visits to bank branches.

Due to the worldwide spread of the COVID-19 pandemic,^2^ there has been an increase in the use of online banking [31]. Such as, Khatun et al. [22] reported that the number of registered mobile banking users in Bangladesh has increased during the pandemic as people’s habits have changed, and they prefer contactless transactions over face-to-face transactions. Therefore, the service quality of online services becomes even more critical in these non-normal situations. The pandemic has created new challenges for banks as most banks have shifted their operations to the online mode, and staff is working from home. Banks have to invest heavily in IT dues to this pandemic to serve their customers better. Frauds have increased as some customers received fraudulent emails from criminals pretending to be bank staff. Banks also have to focus on this issue to ensure the security of their customers. Some older customers who do not know much about online banking tools must be appropriately trained to enhance their satisfaction during this pandemic. “Customer judgment and evaluation of the quality of online services offered by a bank is called online service quality,” as referred by [1, 11].

The Covid-19 pandemic has also affected the world energy and environmental situation. For example, Wang and Su [39] reported that decreasing economic activities due to Covid-19 lockdown directly influenced China energy consumption and further prevented environment from pollution. Wang and Zhang [40] found that there is a spillover effect of China economic recovery after the Covid-19 pandemic on other countries energy consumption i.e., their energy consumption will increase especially in high-income countries, followed by middle-income countries. Wang et al. [41] reported that the US oil consumption during the Covid-19 pandemic was about 18.14% lower than the normal Covid-19 free situation which indicates that there was lesser use of energy resources due to the Covid-19 restrictions.

Amin [1] described “electronic satisfaction as the evaluations of traditional experience with traditional bank services with online customer experience”. Amin [1], and Zeithaml et al. [42] reported that “e-loyalty is the repeated purchase of online services from a bank by online customers. These customers will frequently visit the same bank website or use the mobile app frequently in future for banking services”. The electronic service quality of online banking services needs to be improved as it would help increase the confidence of customers and hence more satisfied customers. This higher electronic satisfaction (e-satisfaction) of customers would lead to higher retention and higher electronic-loyalty [8].

Pakistan is a developing country that is currently facing many economic challenges.^3^ The banking sector has always played an essential role in its economic development. Due to the advancement in technology, many banks have adopted electronic banking services in recent years as these banks are trying to reduce their cost and be more efficient to build better customer relationships. According to the State Bank of Pakistan (SBP), there are 3.1 million online banking customers in Pakistan who enjoy the online banking services of 27 banks online in the country. 8.6 million Transactions totaling 362.3 billion were carried out during the first quarter of 2019 through internet banking. These figures indicate the potential of internet banking in Pakistan. As per SBP policy guidelines, banks in Pakistan have taken several preventive measures during this pandemic to serve their customers better. These measures include the availability of hand sanitizers in branches and ATMs, mandatory mask wearing for banking staff and customers, an awareness campaign about social distancing both through social media and in bank branches, and advice to customers to use the internet and mobile banking during the pandemic to limit their visits to branches, etc. Therefore, the quality of services under online banking must meet the customers’ expectations to develop more satisfied customers with greater loyalty.

Israr et al. [18] explored the indirect effect of customer’s satisfaction on the relationship between service quality and loyalty for Pakistani banking customers of conventional banks. However, the electronic aspects of service quality, service satisfaction, and service loyalty are not covered by this study. Therefore, this study would explore this research gap specifically for Pakistani online banking customers. Further, previous studies have used other mediation techniques like structural equation modeling (SEM). However, the current study used a more sophisticated technique of mediation analysis provided by Preacher and Hayes [32] and Baron and Kenny [4] to make the study findings more robust. Kaya et al. [21] also studied the mediating effect of e-satisfaction on the relationship between e-service quality and e-loyalty but our study is different from their study as we have tested this relationship in non-normal situation like the Covid-19 pandemic which is one of the major contributions to the current literature. We have used different dimensions of e-service quality. Further, this study has used Oliver [29, 30] Expectancy-Disconfirmation Paradigm (EDP) during non-normal situations like the Covid-19 pandemic, which states that customers compare outcomes with expectations and decide about service satisfaction or dissatisfaction. These aspects, we believe, would add theoretical and methodological rigors to this study and turn it to be more scientific. Therefore, this study is aimed to investigate the indirect role of customers' online satisfaction between the association of online service quality and online loyalty of the customers of online banking services provided by conventional banks during the Covid-19 pandemic.

The study is organized as follows for the remaining sections. In “Review of literature” section of the study discusses the relevant literature, especially online banking literature, to formulate hypotheses. In “Research methodology” section sheds light on the study's methodology, including population and sample size, research instrument for data collection, bootstrap method of mediation, and data analysis tools. In “Results and discussion” section presents the study results and relevant discussion supporting theoretical and empirical findings. The last and 5th section concludes the study with practical implications and future research direction.

## Review of literature

There is a vast literature on the relationship between service quality, customer satisfaction, and customer loyalty in various industries, especially the banking industry, but our focus in this study will mainly be on studies related to e-service quality, e-loyalty and e-customer satisfaction on the internet banking.

### Dimensions of e-service quality

According to Amin [1], “customer judgment and evaluation of the quality of online services offered by a bank is called online service quality.” This researcher stressed that the four essential predictors of online service quality are personal user needs, website organization, the efficiency of the website, and user-friendliness. According to Blut [7], website design refers to elements such as information quality, shopping process, the convenience of the website, product selection on the website, price offerings, personalization of the website and website availability. Every online customer experiences these elements. These elements contribute positively to online service quality. An efficient website is transaction-oriented, information-oriented, and customer-oriented [35]. Overall view, outlook and functional organization of the website are called website organization [9]. Several studies found a significant association between website organization and online service quality [1, 13, 34].

According to Grönroos [10], banks must give due importance to users' personal needs as it helps banks acknowledge the lifestyle, sex, age, and preferences of online users and initiate new features to increase users fulfillment. Completing maximum transactions is termed website efficiency in online banking [1, 34]. Amin [1] and Raza et al. [34] found website efficiency significant in online banking service quality.

### E-service quality and e-loyalty

Anderson and Srinivasan [3] stated that “a customer is considered loyal in online banking if he/she is satisfied and involved in the repeated purchase of the service or product from the bank website”. Several researchers reported that the impact of online service quality on the loyalty of online customers is significant and positive. For instance, concerning internet banking, Amin [1] studied the effect of e-service quality on e-loyalty in Malaysian banks by employing customers personal needs, website organization of banks, service friendliness to the customer, and website efficiency as measures of e-service quality. The researcher found that online service quality had a significant positive effect on loyalty of online customers, and all four dimensions of online service quality are essential for keeping customers loyal by applying structural equation modeling (SEM).^4^ Rita et al. [35] found that online service quality dimensions of website design, security, privacy, and fulfillment are essential for Indonesian online customers. Meanwhile, customer behavior is significantly associated with overall e-service quality for these Indonesian customers.

Hsu and Nguyen [15] tested the dimensions of online service quality in Vietnamese customers and found service reliability, service efficiency, design of a website, and fulfillment as significant influencers of online customer satisfaction. They also found that their satisfaction with the service strongly influences the loyalty of these customers. While, Kaur and Kiran [20] also found website interface and access along with convenience and security as significant predictors of online loyalty for Indian banking customers. Based on this literature, we present the following research hypothesis:

#### H_1_

E-service quality positively and significantly influences e-loyalty of online banking customers.

### E-service quality and e-satisfaction

Mofokeng [28] has described e-commerce satisfaction as the emotional reaction of a consumer to the overall transaction experience. Satisfaction as a consequence of service quality is reported by several researchers. Such as, Amin [1] explored the effect of online service quality on online satisfaction for Malaysian banking customers and found their relationship to be significantly positive. Hammoud et al. [11] found that responsiveness, credibility, ease of use, communication, security and privacy, service reliability and efficiency are strong predictors of satisfaction. Similarly, Singh [36] also reported credibility, service efficiency and responsiveness as important influencers of online service quality and online satisfaction for Indian online banking users.

Supriyanto et al. [37], found a significant association between e-service quality and the online satisfaction of banking customers by using similar dimensions of online service quality. Mofokeng [28], who applied the structural equation modeling, indicated that security, information quality, product delivery, and variety of products significantly influence the satisfaction of online buyers. They also reported that information quality and customer satisfaction play an essential role in the loyalty of web stores customers. Several other researchers like Anderson and Srinivasan [3], and Hsu and Nguyen [15] also reported that online service quality is positively related to the satisfaction of online banking users. Keeping in view these studies, we propose the following hypothesis:

#### H_2_

E-service quality has a significant positive impact on e-satisfaction of banking customers.

### E-satisfaction and e-loyalty

Several studies have empirically tested the effect of e-satisfaction on e-loyalty in online banking. For example, Amin [1] studied the effect of e-satisfaction on e-loyalty for Malaysian banking customers and found that the former affects the latter significantly positive. Gera [9] also found a direct effect of e-satisfaction on positive word of mouth and hence e-loyalty. Satisfied customers involve in repeated transactions with online banks as their loyalty toward these banks is very high, as referred by Amin [1]. Mofokeng [28] indicated that customer satisfaction and information quality determine customers’ loyalty to online stores. Based on these studies, the researcher proposes the below hypothesis.

#### H_3_

E-satisfaction has positive influence on e-loyalty of banking customers.

### Mediating effect of e-satisfaction between the association of e-service quality and e-loyalty

The significant amount of the above literature evidence that offering better e-services will ultimately lead the customers to be more satisfied with online services. This greater satisfaction will lead to repeated purchase behavior with the same bank and recommendation of the services to others through positive word of mouth. This will ultimately enhance the loyalty of online customers.

Supriyanto et al. [37] found that better quality of online services results in delighted customers, which ultimately leads to increased loyalty of customers in online banking. Indrasari et al. [17] researched the determinants of user satisfaction and user loyalty in online banking during Covid-19. They found service quality, reliability, application, and website design as significant determinants of user online satisfaction and loyalty. They further found that security and privacy affect online user loyalty and not online satisfaction. Expectancy theory suggests that consumers’ motivation depends on predicted outcomes’ perceived value [33]. This improves consumers’ motivation toward satisfaction and loyalty since it may help bank managers develop customers’ attributes toward loyalty through satisfaction in online banking. Online banking customers will be motivated if they believe that perceived values (expectancy) lead to e-satisfaction (instrumentality), and e-satisfaction will lead to customer e-loyalty (valence). Based on the above rationale, we suggest the following mediation hypothesis:

#### H_4_

E-Satisfaction mediates the relationship between e-service quality and e-loyalty of banking customers.

This study has three major contributions to the relevant literature. First, this study has used a more sophisticated technique of mediation analysis provided by Preacher and Hayes [32] and Baron and Kenny [4] jointly in non-normal situation like the Covid-19 pandemic to make the study findings more robust and scientific. Secondly, this study has used Oliver [29, 30] Expectancy-Disconfirmation Paradigm (EDP) during non-normal situations like the Covid-19 pandemic. Thirdly, this is one of the pioneer studies in non-normal situation in an emerging economy like Pakistan where e-commerce is still in its very early stages.

### Conceptual model

**Fig. 1:**
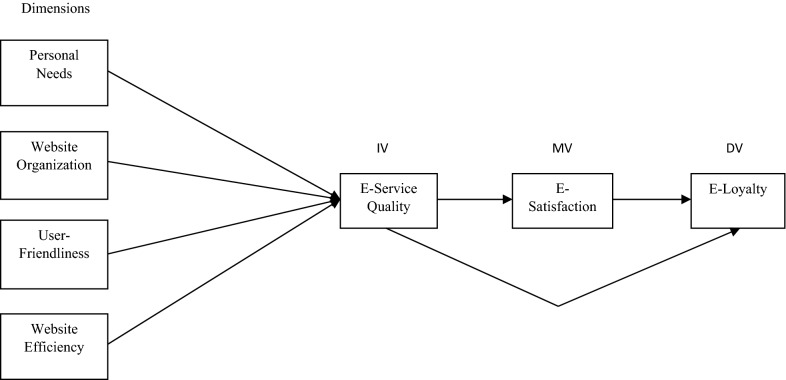
Conceptual framework of study variables

Oliver [29, 30] proposed the Expectancy-Disconfirmation Theory. According to this theory, customers compare product outcomes with their expectations from the product. Confirmation occurs if outcomes meet the expectations. A positive disconfirmation occurs if performance (outcomes) exceeds expectations (satisfied customers). A negative disconfirmation occurs if expectations exceed performance (outcomes) (dissatisfied customers). If the perceived performance of the online service is equal to customer expectations, then a simple confirmation occurs. The customer should also be satisfied in this situation. A satisfied customer will repeat the purchase of services from the same bank, and hence e-loyalty will increase. This is one of the prominent theories of customer satisfaction used primarily in typical situations, but this study has used this theory in non-normal situations like the Covid-19 pandemic, where a better quality of online services is needed. Furthermore, this study is more innovative in the sense that it has used Preacher and Hayes [32] mediation and Baron and Kenny [4] technique jointly in non-normal situation like the Covid-19 pandemic which makes the study more rigorous and its findings more scientific. Based on the previous literature, the following conceptual framework has been developed and presented in Fig. 1.

## Research methodology

### Population and sample of the study

The study population is Online (Internet) banking customers in Pakistan. A convenience sampling technique is used for sample selection as a sampling frame was unavailable for the probability sampling. A self-administered questionnaire is used for data collection by personally visiting the customers and various branches of banks in cities like Karachi, Lahore, Islamabad, Peshawar, Abbottabad, Quetta, Bannu and Sargodha of Pakistan. Rationale for selecting these districts is that most of the customers of these districts use online banking services compared to other districts of Pakistan. Further, these districts have been affected very severely as the Covid-19 positivity ratio is very high (above 10%) during the study period, and most of the customers prefer to use online banking services than traditional face-to-face banking services during this period of Covid-19. The researchers have distributed eight hundred questionnaires to the online banking users, out of which 442 were returned, resulting in a response ratio of 55.25. The low response rate is due to Covid-19 situations as customers were reluctant to return questionnaires due to social distancing. The researchers have used Krejcie and Morgan [24] technique for sample size calculation in this study. This sample size is feasible as Amin [1] also reports a similar sample size.

## Research design instrument

This research is empirical and primary. A regression, correlational and quantitative research design is used to investigate the relationship between the various variables of the study for online banking customers in Pakistan.

This study has employed the instrument used by Amin [1], which reported that the significant predictors of online banking service quality are users personal needs, organization of the website, user-friendliness, and website efficiency. Three items are used for personal need, four for website organization, four for user-friendliness, and three for website efficiency [13, 14]. E-satisfaction is measured by five items [1]. Similarly, five items were used to measure e-loyalty [1]. Amin et al. [2] have also employed these items for e-loyalty in internet banking. The questionnaire was suitably adapted for Pakistani online banking customers.

A five-point Likert scale is used to measure our independent variable e-service quality, and dependent variable e-loyalty, ranging from strongly disagree (1) to strongly agree (5). A similar scale with strongly dissatisfied (1) to strongly satisfied (5) has been used for the study mediator, i.e., e-satisfaction. This research has adopted the five-point Likert scale of [1]. There are several reasons of using a five-point Likert scale like it is simple and easy to understand, it takes less time to fill out the questionnaire, it increases response rate, fits well in Smartphone screens and it is standard (universal) tool of collecting data. The demographics of the respondents are given in Table 1. Most of the respondents are aged below 30 years (96.6%) which shows that most young customers use internet banking services as they are more familiar with technology than aged banking customers.

**Table 1:** Characteristics of the respondents

Demographics	Classification	Frequency	Percentage
Gender	Female	178	40.3
	Male	264	59.7
Age	Below 30	427	96.6
	30–45	10	2.3
	45–60	2	0.5
	Above 60	3	0.7
Marital status	Married	419	94.8
	Single	14	3.2
	Separated	9	2.0
Education	High school	5	1.1
	College	159	36.0
	Master	272	61.5
	P.hD	6	1.4
Income	Below 40,000	256	57.9
	41,000–50,000	79	17.9
	51,000–70,000	19	4.3
	Above 70,000	88	19.9

This table shows the number and percentage of participants of different age groups, male and female participants, married, unmarried and separated participants, participant’s distribution based on various educational degrees, and participant’s distribution based on different income levels

The majority of the respondents are males (59.7%), which suggests that most men prefer to use technology to carry out banking transactions in a male-dominated society like that of Pakistan. 94.8% of the respondents are married, which proves those already settled customers with families prefer to use online banking services as they have to meet particular financial demands of their families. Most of the customers are university graduates (61.5%), which shows that most educated people use online banking services as they know these services. The majority (57.9%) of the respondents have an income level below Rs-30,000, which shows that most middle class of Pakistan is interested in online banking services.

### The bootstrap method of mediation

Preacher and Hayes [32] meditation technique, a linear programming technique, is used to test the mediational effect of e-satisfaction on the relationship between e-service quality and e-loyalty in online banking customers of Pakistan. This is a non-parametric resembling test, and it does not rely on normality assumption, hence, it is also suitable for small samples. Among revolutionary models of assessing mediation such as Baron and Kenny [4], Hyman [16], Judd and Kenny [19], MacCorquodale and Meehl [24], MacKinnon and Dwyer [25], MacKinnon, Warsi, and Dwyer [27], MacKinnon et al. [26] and Preacher and Hayes [32], the researchers used more sophisticated method of assessing mediation to inculcate rigorous assessment of mediation in this study.

### Baron and Kenny [4] mediation technique

Baron and Kenny [4] have suggested four steps for mediation analysis. First step (first condition) involves the causal impact of independent variable on the mediator. This step has been termed as path a by Baron and Kenny [4] and this affect must be significant. Second step (second condition) involves the significant impact of independent variable on the dependent variable and it is termed as path c. Third step (third condition) involves the impact of mediator on the dependent variable and is termed as path b. Fourth step (fourth condition) involves the influence of independent variable on the dependent variable in the presence of mediator and it is termed as path c prime. Full mediation occurs if the effect of independent variable on dependent variable becomes insignificant in fourth step. Partial mediation occurs if the impact of independent variable on dependent variable has reduced but is still significant in step four.

### Data analysis techniques

SPSS-21 software is used for analyzing the data in the following sequence. In order to avoid incomplete and inappropriate responses, all the questionnaires were carefully scrutinized. Descriptive analysis was done to judge the nature of the data. To identify the relationship between various variables, correlation analysis was carried out. Finally, regression analysis was performed to see the cause and effect relationship of variables.

## Results and discussion

### Descriptive and reliability analysis

Descriptive (minimum, maximum, mean, standard deviation) and reliability analysis are given in Table 2. Respondents, on average, have scored 3.9706, 3.7036 and 3.7602 on e-loyalty, e-loyalty and e-satisfaction, respectively. Data are standard as shown by minimum and maximum values for each variable. Reliability analysis indicates that Cronbach Alpha of all variables is above the standard of 0.70 (see Table 2).^5^ It shows that the scales used are reliable and that our data set passes the reliability test.

**Table 2:** Reliability analysis and descriptive

Variable	Min	Max	Mean	Std	Cronbach’s alpha
E-loyalty	1.00	5.00	3.9706	0.7008	0.772
E-services quality	1.00	5.00	3.7036	0.6636	0.701
E-satisfaction	2.00	5.00	3.7602	0.6034	0.768

This table shows the descriptive analysis i.e., minimum, maximum, and mean, standard deviation as well as Cronbach’s Alpha of the study variables

### Correlation analysis

Table 3 shows the results of the Pearson correlations coefficient between the study variables. Association between e-service quality and e-loyalty is significantly positive at a significance level of 1% percent (*r* = 0.279** with *p* ≤ 0.01), which support our first hypothesis (H_1_). There is significant positive relationship between e-service quality (independent variable) and e-satisfaction (mediator) (*r* = 0.405** with *p* ≤ 0.01) at significance level of 1%. This supports our second research hypothesis (H_2_). The study mediator e-satisfaction is significantly and positively related to the dependent variable e-loyalty at a 1% significance level (*r* = 0.573** with *p* ≤ 0.01). This provides support to our third hypothesis (H_3_).

**Table 3:** Pearson correlations analysis

Variables	E-loyalty	E-service quality	E-satisfaction
E-loyalty	1		
E-service quality	0.279**	1	
E-satisfaction	0.573**	0.405**	1

**Correlation is significant at 0.01 level (2-tailed)

### Regression analysis

#### Regression analysis through Preacher and Hayes [31] technique

Regression results by applying Preacher and Hayes [32] technique have been generated using SPSS 21. Its analysis and interpretations are given below.

Regression analysis by Preacher and Hayes [32] has been used to determine the mediating effect of e-satisfaction between the relationship of online service quality and online loyalty. Panel “A” of Table 4 (path a) shows that our independent variable e-service quality is significantly and positively affecting our mediating variable e-loyalty, *β* = 0. 4459, SE = 0. 0407, *p* < 0.05, (path b) indicates that the mediator e-satisfaction positively influences the dependent variable e-loyalty of online banking customers, *β* = 0. 7312, SE = 0. 0508, *p* < 0.05. Hence the study mediation hypothesis is supported by these results. The path c proves that our dependent variable is significantly and positively affected by e-service quality (*β* = 0. 4455, SE = 0. 0525, *p* < 0.05). When the mediator is introduced in the model (path cʹ), e-loyalty is still positively and significantly influenced by online service quality but with lesser strength, as shown by coefficient (beta = β) decline from 0.4455 to 0.1195 with probability (*p*-value) 0.0000, which is less than 0.05, in line with the mediation hypothesis.

**Table 4:** Regression analysis by Preacher and Hayes [32] technique

Panel A: Mediation analysis					
#	Variable	Coefficient (*β*)	SE	*t*-value	*p*-value
1	IV to Mediator (a path)				
	E-satisfaction	0.4459	0.0407	10.9680	0.0000
2	Direct effects of mediator on DV (b path)				
	E-satisfaction	0.7312	0. 0508	14.3846	0.0000
3	Total effect of IV on DV (c path)				
	E-service quality	0.4455	0.0525	8.4836	0.0000
4	Direct effect of IV on DV (cʹ path)				
	E-service quality	0.1195	0.0489	2.4435	0. 0149

Panel B: Summary of model for DV model					
*R*^2^	Adjusted *R*^2^	*F*	*df*1	*df*2	*p*-value
0.4159	0.4132	156.2841	2.0000	439.0000	0.0000

Bootstrap outcome for indirect effects				
Panel C: Indirect effects of IV on DV through Mediator (ab paths)				
	Data	Boot	Bias	SE
Total	0.3260	0.3237	− 0.0023	0.0512
E-Satisfaction	0.3260	0.3237	− 0.0023	0.0512

Panel D: Bias corrected confidence intervals		
	Lower	Upper
Total	0.2384	0.4441
E-satisfaction	0.2384	0.4441

Panel “A” of this table shows the significant effect (*p* < 0.05) of e-service quality on e-satisfaction(path a), significant effect (*p* < 0.05) of e-satisfaction on e-loyalty(path b), significant effect(*p* < 0.05) of e-service quality on e-loyalty (path c) without the mediator e-satisfaction, and significant effect(*p* < 0.05) of e-service quality on e-loyalty (path cʹ) in the presence of mediator e-satisfaction. Panel “B” indicates that the model is a good fit and significant (*p* < 0.05). Whereas, Panel “C” indicates that e-service quality is linked with approximately 0.3260 points higher e-loyalty scores mediated by e-satisfaction. It shows the indirect effects. Moreover, panel “D” indicates the lower and upper confidence limits, and it shows that the value zero does not fall between the lower and upper confidence limits of 0.2384 and 0.4441, respectively, which supports significant mediation as per [32].

Level of confidence: 95

N0 of bootstrap resamples: 1000

*DV* e-loyalty, *IV* e-service quality, *MED* e-satisfaction, Sample Size = *n *= 442

Panel “B” of this table shows that approximately 41.59% of the variance in a dependent variable, i.e., e-loyalty, was accounted for by the predictors (*R*^2^ = 0.4159), and the model is a good fit and significant with *F* = 156.2841 and *P*-value < 0.05. The indirect influence is tested using the bootstrap estimation approach with 1000 samples. In Panel “C”, the result shows that the indirect coefficient is positive and significant, *β* = 0.3260, SE = 0.0512, 95% CI = 0.2384, 0.4441. E-service quality is linked with approximately 0.33 points higher e-loyalty scores as mediated by e-satisfaction. Panel “D” indicates that the value zero (0) does not fall between lower confidence and upper confidence limits of 0.2384 and 0.4441, respectively, which supports significant mediation by e-satisfaction between e-service quality and e-loyalty of online banking customers.

#### Regression analysis through Baron and Kenny [4] technique

The researchers have used Baron and Kenny [4] mediation in order to find the mediating effect of e-satisfaction on the relationship between e-service quality and e-loyalty. This Technique along with Preacher and Hayes [32] will lead to more robust findings of the study. Preacher and Hayes [32] technique only tell about whether there is any mediation or not. Baron and Kenny [4] go one step further by showing whether the mediation is full or partial.

Baron and Kenny [4] suggested four conditions (four models) to test mediating effect of any variable between independent variable and dependent variable.

##### Condition 1

The IV should significantly influence the mediator.

We have tested this condition and the result is presented under model 1 of Table 5. Condition 1 of Baron and Kenny [4] is satisfied as e-service quality (IV) significantly and positively influences e-satisfaction (mediator) (*β* = 0. 369, SE = 0. 040, *t*-value = 9.302, *p* < 0.05). One unit increase in e-service quality increases e-satisfaction by 0.369 units. Standard error (SE) is sufficiently small (0.040) indicating that the selected sample belongs to the given population and is sufficiently large. The regression model is a good fit and significant at 1% level with *F* = 86.531.

**Table 5:** Mediation analysis by Baron and Kenny [4]

Variable	Coefficient (*β*)	SE	*t*-value	*p*-value
*Model 1: Regression Result Between E-Service Quality (IV) and E-Satisfaction (Mediator)*				
E-SQ	0.369	0.040	9.302	0.000*
*R*^2^ = 0.164, *F* = 86.531*				
*Model 2: Regression Result Between E-Service Quality (IV) and E-Loyalty (DV)*				
E-SQ	0.294	0.048	6.086	0.000*
*R*^2^ = 0.078, *F* = 37.039*				
*Model 3: Regression Result Between E-Satisfaction (Mediator) and E-Loyalty (DV)*				
E-SAT	0.666	0.045	14.669	0.000*
*R*^2^ = 0.328, *F* = 215.171*				
*Model 4: Regression Result Between E-Service Quality (IV) and E-Loyalty (DV) in the presence of E-Satisfaction(Mediator)*				
E-SQ	0.059	0.045	1.298	0.195
E-SAT	0.640	0.050	12.893	0.000*
*R*^2^ = 0.331, *F* = 108.595*				

*Indicates significance at 1% level. This table indicates the various steps of Baron and Kenny [4] mediation analysis in four different models. Regression model 1 indicates the impact of e-service quality on e-satisfaction. Regression model 2 indicates the influence of e-service quality on e-loyalty. Regression model 3 indicates the affect of e-satisfaction on e-loyalty, and model 4 shows the influence of e-service quality on e-loyalty in the presence of e-satisfaction (mediator)

##### Condition 2

The independent variable significantly affects the dependent variable.

Model 2 in Table 5 indicates that e-service quality (IV) positively and significantly affects e-loyalty of banking customers (*β* = 0. 294, SE = 0. 048, *t*-value = 6.086, *p* < 0.05) which satisfies Baron and Kenny [4] condition 2. An increase in one unit in e-service quality enhances e-loyalty by 0.294 units which is significant at 1% level (*p* < 0.05). The SE (0.048) is again small indicating that the sample size is large and sufficient. The regression model is a good fit and significant at 1% level with *F* = 37.039.

##### Condition 3

Mediator significantly affects the dependent variable.

Model 3 in Table 5 shows that our mediator e-satisfaction significantly and positively influence e-loyalty (DV) (*β* = 0. 666, SE = 0. 045, *t*-value = 14.669, *p* < 0.05) and it confirms the third condition of Baron and Kenny [4] mediation analysis. An increase in one unit in e-satisfaction (mediator) increases e-loyalty (DV) by 0.666 units which is significant at both 5% and 1% level (*p* < 0.05). Regression model 3 is also significant with *F* = 215.171 at 1% level and sample size is sufficiently large (lower SE = 0.0450).

##### Condition 4

The impact of e-service quality (IV) on e-loyalty (DV) reduces after the introduction of e-satisfaction (Mediator) in the regression model.

Model 4 in Table 5 indicates that the effect of e-service quality (IV) on e-loyalty (DV) becomes insignificant at 5% as *p*-value is 0.195 (*β* = 0.059, SE = 0. 045, *t*-value = 1.298) after the introduction of e-satisfaction (mediator) in the model. In fact the regression coefficient for e-service quality (IV) has reduced from 0.294 to 0.059 after the inclusion of e-satisfaction (Mediator) in the model with *p*-value less than 0.05 indicating full mediation by e-satisfaction. Regression model 4 is also a good fit and significant at 1% level with *F* = 108.595.

The study of Kaya, et al. [21] reported partial mediation in normal situations while our study reports full mediation in non-normal situation like the Covid-19 pandemic. Application of Baron and Kenny [4] mediation has confirmed the results of Preacher and Hayes [32] technique which leads to more robust findings for the study in non-normal situation like the Covid-19 pandemic.

## Summary of hypotheses

Table 6 presents a snapshot of the four hypotheses developed in the literature review. It can be confirmed that the relevant results of the study support all four hypotheses.

**Table 6:** Summary of hypothesis

Hypothesis	Description	Results
Hypothesis 1	E-service quality has a significant positive influence on e-loyalty	Accepted
Hypothesis 2	E-service quality has a significant positive relationship with e-satisfaction	Accepted
Hypothesis 3	E-satisfaction has a significant positive relationship with e-loyalty	Accepted
Hypothesis 4	E-satisfaction mediates the relationship between e-service quality and e-loyalty	Accepted

This table shows that our results support all the four hypotheses of the study and e-satisfaction act as a mediator between quality of online services and the loyalty toward these services

The study findings show that online service quality has a significant positive effect on the e-loyalty of online banking customers, which shows that H_1_ is accepted. It means that better services like internet banking and mobile banking enhance customers' repurchase behavior, and customers do not shift to other banks as they remain loyal. User personal needs, website organization, the efficiency of a website, and user-friendliness dimensions of online service quality become even more critical in non-normal situations like the Covid-19 pandemic as customers consider it more important to evaluate online banking service quality. It will ultimately shape their future behavior regarding banking (continue with the same bank, i.e., remain loyal to the bank or shift to any other bank with a better quality of online services, i.e., change e-loyalty). This finding is consistent with the finding of [1, 35]. E-service quality positively affects e-satisfaction, so our H_2_ is also supported. It shows that good quality of online services leads to more satisfied online banking customers. Customer satisfaction, customer value, trust, and commitment can be enhanced by improving online service quality, which is essential for firms’ long-term survival, and this finding is similar to the study of [20, 35]. Expectancy-Disconfirmation Theory of Oliver [29, 30] exclusively support this finding of the study.

The result supports H_3_, which testifies that e-satisfaction of online customers significantly and positively affects the e-loyalty of these customers. Higher satisfaction of these online customers results in a higher level of loyalty and commitment to the bank they are engaged with. This argument is supported by the study of Mofokeng [28]. Dissatisfied customers usually switch to other banks because the service performance does not meet their expectations. This result is similar to the study of [1, 5]. Their study results also show that e-satisfaction of online customers is playing an indirect role between the association of e-service quality and e-loyalty. This indirect effect supports our meditational hypothesis (H_4_). This view is consistent with the Expectancy-Disconfirmation Paradigm (EDP) of Oliver [29, 30], a theory for measuring customer satisfaction from the perceived quality of service. If banks give due importance to customers personal needs, security, website efficiency, and design a well-organized website (dimensions of e-service quality in this study) that is user friendly, then online banking customers will perceive the services quality of such banks to be of premium nature and hence they will be more satisfied as the service performance will match or exceed their expectations. It will lead to trustworthy customer relationships, and they will not shift to other banks and remain loyal to the current bank. Therefore, we can conclude that banks' better quality of online services will enhance their customer online satisfaction, which paved the way for enhanced service use frequency of the bank, intention to recommend the bank to others, and repeated purchase from the bank are signs of e-loyalty.

This study is different from past studies. For example, Vun et al. [38] study found partial mediation of customer satisfaction between the service quality dimensions of efficiency, privacy and responsiveness and customer loyalty while our work supports full mediation. Similarly, Ul Haq and Awan [12] study also found only a partially mediating effect of online banking satisfaction between service quality dimensions of reliability and website design with online banking loyalty.

## Conclusion, practical implications and future study direction

### Conclusion

The study serves two purposes. The first one is to check the impact of online services quality on the loyalty of online banking customers, particularly during the Covid-19 pandemic. The second one is to see whether the satisfaction of online banking customers plays any mediating role in their relationships during the Corona Virus pandemic. We found a significant positive effect of e-service quality on the e-loyalty of online banking customers in Pakistan during the Covid-19 pandemic. Like other service sectors, the banking sector has also shifted its services from traditional to online ones as the government has imposed multiple lockdowns and people were reluctant to visit bank branches physically. The districts selected for the study have been affected very severely as the positivity ratio is above 10%, and banking customers avoid face-to-face contact in these non-normal situations. They prefer banks' online services, and hence its quality matters a lot to them during these unusual situations. Covid-19 pandemic has increased the need for online banking services, and customers now give more importance to the quality of these services as they play a significant role in customer satisfaction or dissatisfaction.

We further found that better online services enhance the satisfaction level of online banking customers, leading to improved loyalty and commitment levels of these customers. The study also found a fully mediating effect of e-satisfaction on the association of online service quality and online loyalty for banking customers in non-normal situations like the Covid-19 pandemic, proving the meditational hypothesis, one of this study's main contributions while using the approach as introduced by [32] and Baron and Kenny [4]. As frauds have increased during this pandemic (several customers received emails and calls pretending to be bank staff while actually, they were criminals), banks must give due importance to the security and privacy of their customers as these are the important aspects of service quality. This study is unique because it has used Expectancy-Disconfirmation Theory of Oliver in non-normal situations like the Covid-19 pandemic where most sectors, including the significant banking sector, have shifted their operations to online mode. Moreover, as rigor is desired in every scientific research, the researchers added methodological in this study to guide future studies. Among various models of assessing mediation, the researchers used Baron and Kenny [4] and Preacher and Hayes [32] sophisticated method to assess mediation during non-normal situations like the Covdi-19 pandemic. This technique can be used even in small sample size, making it preferable to other mediation techniques.

The study findings add scientific value as they are applicable to the banking sector in particular in non-normal situations like the Covid-19 pandemic and the overall service sector in general. Further, two different methods of mediation have been employed and this makes the study more rigorous and scientific.

The study contributed to the relevant literature in three different ways. First, this study has used a more sophisticated technique of mediation analysis provided by [32] and Baron and Kenny [4] jointly in non-normal situation like the Covid-19 pandemic to make the study findings more robust and scientific. Secondly, this study has used [29, 30] Expectancy-Disconfirmation Paradigm (EDP) during non-normal situations like the Covid-19 pandemic. Thirdly, this is one of the pioneer studies in non-normal situation in an emerging economy like Pakistan where e-commerce is still in its very early stages.

### Practical implications

Implications of this study are divided into three categories. First, banks must improve the quality of online services, resulting in enhanced customer satisfaction and commitment, as this is also a requirement during the Covid-19 pandemic. Second, banks websites must be well organized and user-friendly, leading to e-loyalty of customers. Thirdly, management must ensure the security of the bank website and, most importantly, the mobile application used by the majority of the customers for online banking. Customers who are old aged and do not know about the use of online banking must be adequately trained so that their satisfaction is enhanced.

### Future study direction

This study is limited to conventional banking only. Future studies can expand it to Islamic banking and compare conventional and Islamic banking customers in terms of online aspects of service quality, loyalty and the indirect role of online satisfaction between their relationships. Future studies may use a moderator like e-commerce experience between the relationship of e-service quality and e-loyalty of online banking customers.

## Data Availability

The data used during the current study are of primary nature obtained by personally visiting bank branches in cities like Haripur, Abbotabad, Bannu, Bhakkar, and Sargodha. A total of 450 questionnaires were distributed among the online banking customers and 442 were returned.

## Abbreviations

E-Service: Electronic services
E-Quality: Electronic quality
E-Loyalty: Electronic loyalty
E-Satisfaction: Electronic satisfaction
IT: Information technology
SBP: State bank of Pakistan
SEM: Structural equation modeling
EDP: Expectancy-disconfirmation paradigm
Covid-19: Corona Virus pandemic
ATMs: Automated teller machine

## Footnotes

1 In the United States of America, sales increased for e-commerce provider grocery stores by 16% and non-store retailers by 14.8%. In Europe, retail sales via the internet increased by 30% in April 2020 compared to April 2019.

2 Total confirmed cases 274.7 million, Total reported deaths are 5.3 million, Daily deaths 4621, and daily confirmed cases 480,835 (*Source*: Jhon Hopkins University, National Public Health Agencies, 20 Dec, 2021).

3 The main economic challenges that Pakistan is facing are rising inflation, rising unemployment and poverty, rising external and circular debt, low foreign investment and high fiscal deficit.

4 Structural equation modeling is a powerful mediation technique to examine multivariate causal relationships between variables. It can test both direct and indirect effects on pre-assumed causal relationships, which is why it is different from other techniques.

5 Cronbach alpha values of 0.7 are considered a standard and acceptable level of internal consistency.
